# Electromagnetic Field Drives the Bioelectrocatalysis of γ-Fe_2_O_3_-Coated *Shewanella putrefaciens* CN32 to Boost Extracellular Electron Transfer

**DOI:** 10.3390/ma17071501

**Published:** 2024-03-26

**Authors:** Xiaohai Wang, Zhuanzhuan Shi, Zhikai Wang, Xiaoshuai Wu

**Affiliations:** Institute of Materials Science and Devices, School of Materials Science and Engineering, Suzhou University of Science and Technology, Suzhou 215011, China; wangxiaohai0316@163.com (X.W.); jasonwang01033@163.com (Z.W.)

**Keywords:** magnetic nanomaterial, electromagnetic field, bioelectrocatalysis, extracellular electron transfer, *Shewanella putrefaciens*

## Abstract

The microbial hybrid system modified by magnetic nanomaterials can enhance the interfacial electron transfer and energy conversion under the stimulation of a magnetic field. However, the bioelectrocatalytic performance of a hybrid system still needs to be improved, and the mechanism of magnetic field-induced bioelectrocatalytic enhancements is still unclear. In this work, γ-Fe_2_O_3_ magnetic nanoparticles were coated on a *Shewanella putrefaciens* CN32 cell surface and followed by placing in an electromagnetic field. The results showed that the electromagnetic field can greatly boost the extracellular electron transfer, and the oxidation peak current of CN32@γ-Fe_2_O_3_ increased to 2.24 times under an electromagnetic field. The enhancement mechanism is mainly due to the fact that the surface modified microorganism provides an elevated contact area for the high microbial catalytic activity of the outer cell membrane’s cytochrome, while the magnetic nanoparticles provide a networked interface between the cytoplasm and the outer membrane for boosting the fast multidimensional electron transport path in the magnetic field. This work sheds fresh scientific light on the rational design of magnetic-field-coupled electroactive microorganisms and the fundamentals of an optimal interfacial structure for a fast electron transfer process toward an efficient bioenergy conversion.

## 1. Introduction

Microbial fuel cells (MFCs) are bioelectrochemical systems that convert chemical energy into electrical energy using electroactive microbes (EAMs) as catalysts [1], representing a clean energy technology [2]. Moreover, they can be used to treat waste/wastewater and have been widely investigated due to their dual efficacy [3,4]. EAMs can utilize an electrode as the terminal electron acceptor for reduction, with the electrode serving as either the electron donor or acceptor depending on whether it functions as the anode or cathode [5,6]. They possess several extracellular electron transfer (EET) strategies for anaerobic respiration, including a direct electron transfer (DET) mediated by outer membrane C-type cytochromes (OM c-Cyts) [7] and nanoconductors [8,9], and an indirect electron transfer (IET) mediated by exogenous or endogenous electron mediators. However, limited by the inefficient EET process and the slow transmembrane process, the power density of MFC is far from reaching the levels of industrial application [10,11]. Hence, there lies a pressing need to construct a simple and highly efficient approach that expedites the EET process.

With the burgeoning advancement in the field of nanoscience, the use of nanomaterial for MFC modification has received widespread attention from researchers and has achieved significant results [12]. A lot of studies have proven that the implementation of functional nanomaterials can significantly reduce charge transfer resistance, leading to a notable enhancement in microbial colonization and biofilm growth [13,14,15]. Additionally, this innovative approach has demonstrated a considerable potential for improving the efficiency of electron transfer to extracellular receptors [16]. Furthermore, highly conductive nanomaterials can serve as electron shuttle channels, which can greatly improve the efficiency of an EET [17]. In these contexts, researchers have applied various advanced nano-functional materials, including metal oxides, carbon-based nanomaterials, metal-based nanomaterials, conductive polymers, and their composites, to the study of MFC [18]. Initially, researchers used a single or composite nanomaterial to modify the anode surface to increase the surface area for bacterial growth and optimize the electrode surface properties [19]. Nonetheless, a noteworthy observation has emerged, revealing that within the natural biofilm formed on the anode surface, a majority of bacteria are located at a considerable distance from the functional nanomaterial. Consequently, only the bacteria inhabiting the innermost layer of the biofilm maintain direct contact with the nanomaterials positioned on the electrode surface [20]. The efficiency of EET is improved only to a limited extent by relying on slow electron jumps in redox centers for electron transfer between bacteria [21]. To address these issues, researchers then proposed the strategy of using nanomaterials hybridized with biofilms [22]. Through a disordered mixed contact of functional nanomaterials with bacteria inside biofilms, the nanomaterials can help facilitate long-distance electron transfer, further improving the efficiency of EET [23]. However, the establishment of a tightly coupled and efficient pathway for electron transfer between EAMs and conductive non-biological surfaces continues to be elusive. To exploit the active sites on the bacterial outer membrane, researchers have proposed the use of nanomaterials to modify the surface or interior of bacteria [24,25,26,27]. The efficient construction of the microbial–nanomaterial interface can optimize the EET efficiency and enhance the power generation performance of MFC. As an example, the strategic utilization of the carbon particle point modification on bacterial surfaces has demonstrated its ability to enhance bacterial adhesion and facilitate the formation of biofilms. Owing to the presence of surface carbon particle points, the maximum current and power output are increased by 7.34 times each, and the EET efficiency is improved [28]. Silver nanoparticles successfully introduced into the transmembrane and outer membrane dramatically enhance the charge extraction rate of MFC, achieving a maximum current density of 5 mA/cm^2^ and a power density of 0.66 mW/cm^2^ [29]. The concept of a single-cell electron collector has been proposed. The team utilized dopamine in situ polymerization on the surface of *S. oneidensis* MR-1 cells to form a primary electron collector, followed by the further assembly of a more efficient electron collector through FeS NPs biomineralization. The remarkable electron transfer rate and electron recovery efficiency have led to a record-breaking bioelectricity generation in *S. oneidensis* MR-1, achieving an impressive power output of 3.21 W/m^2^ [30].

Magnetic nanoparticles (MNPs), regarded as a pivotal class of functional nanomaterials, have garnered extensive attention owing to their remarkable nanoscale characteristics and distinctive magnetic properties, thus triggering substantial research endeavors. Among the MNPs, magnetic hematite (γ-Fe_2_O_3_) is considered one of the most ideal materials for various applications due to its inherent biocompatibility, oxidative stability, high surface area, and good magnetism [31]. Also, *Shewanella* belongs to the group of dissimilatory metal-reducing bacteria with a unique EET behavior, using iron oxides as terminal electron acceptors to complete metabolic and electron transfer processes. Moreover, the trivalent iron ions in magnetic hematite have reducibility, which is an important electron carrier property for OM c-Cyts and iron oxide proteins. However, there are currently a few reports on the application of γ-Fe_2_O_3_ for the cell surface modification of MFC power generation bacteria.

At the same time, recent investigations have demonstrated that the application of a magnetic field (MF) can facilitate the rapid proliferation of biofilms, enhance the electrochemical activity of power-producing microorganisms, shorten startup time, improve open-circuit voltage, reduce reactor resistance, and enrich power-producing bacteria [32,33,34]. Therefore, the application of an MF in MFCs has been extensively studied. A reduced startup time and an enhanced biofilm electrocatalytic activity were observed with the application of a 100 mT magnetic field to a single-chamber microbial fuel cell using mixed wastewater [35]. A range of MFCs, including single-chamber, double-chamber, and three-electrode cell designs, were fabricated utilizing pure cultured *Shewanella*. Remarkably, under the influence of MF, high voltage outputs were obtained consistently across all configurations. The study revealed that the application of an MF stimulation resulted in an enhanced secretion of mediators and improved catalytic activity. As a consequence, the electron exchange efficiency was markedly improved [36]. In addition, research has indicated that an appropriate magnetic field intensity can increase power generation and reduce internal resistance, while a stronger magnetic field can suppress MFCs performance [37].

In addition to the static magnetic field mentioned above, researchers have also introduced an electromagnetic field (EMF). An EMF can serve as a driving force for controlling the metabolic kinetics of EAMs, and a constant high-intensity magnetic field can inhibit microbial metabolism and normal growth [38]. Therefore, the short-term intermittent application of a magnetic field can have a good regulatory effect on bioelectrocatalysis. Pulse electromagnetic fields (PEMFs) can enhance the enrichment of exoelectrogenic bacteria and accelerate extracellular electron transfer, thereby improving the power generation efficiency. A PEMF causes changes in microbial community and uniformity, leading to a decrease in the microbial diversity of the biofilm [39]. Applying a 2 mT solenoid magnetic field (SOMF) in an osmotic microbial fuel cell (OMFC) increased the coulomb efficiency in a study by 20–30%, producing a current density of 26.58 ± 12 mW m^−2^, power density of 266.29 mA m^−2^, and shortening startup time by 1–2 days. However, performance was reduced when the electromagnetic flux of the coil was increased to 3 mT [40]. In particular, the synergistic application of MNPs and a MF exhibits tremendous potential in enhancing bioelectrochemical electricity generation, facilitating the production of high-value byproducts and efficiently removing pollutants from wastewater sludge [41,42,43]. However, there are still a few reports on the bioelectrochemical system coupling controllable electromagnetic fields with magnetic nanoparticles.

**Figure 1:**
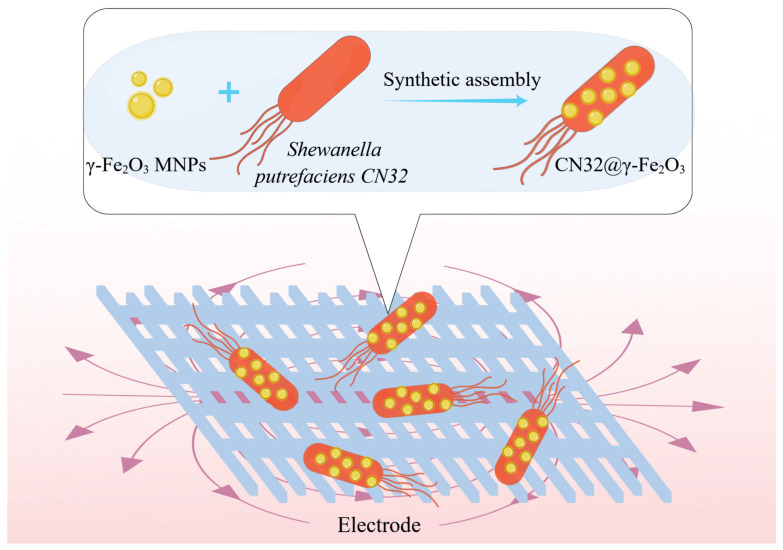
Schematic diagram of the coupling EMF of *S. putrefaciens* CN32 modified with γ-Fe_2_O_3_ MNPs.

In this study, the magnetic nanomaterials γ-Fe_2_O_3_ and *S. putrefaciens* CN32 were self-assembled to form hybrid bacteria CN32@γ-Fe_2_O_3_ (Figure 1). The magnetic nanoparticles in the hybrid bacteria can serve as electron shuttle media and be coupled with electromagnetic fields to construct an MNPs hybrid bacteria-coupled electromagnetic field system to accelerate the bioelectrocatalytic process at the interface, opening up new channels for electron transfer and to improve the EET transfer efficiency and power generation performance. The experimental findings unequivocally demonstrated that the magnetic nanomaterials on the surface of the bacteria enhanced the DET mediated by OM c-Cyts and significantly improved the efficiency of the bacterial and interface EET processes. Under magnetic field stimulation conditions, electrochemical results showed that the MFC system constructed by CN32@γ-Fe_2_O_3_ had a smaller peak spacing, a more negative anodic peak potential, a larger oxidation–reduction area, and a lower charge transfer resistance compared to controls. This experiment provides an uncomplicated, efficient, and low-cost technique for modifying *S. putrefaciens* CN32 using magnetic nanomaterials, which can promote the EET process and has potential value for BES.

## 2. Experimental Methods

### 2.1. Materials and Chemicals

*S. putrefaciens* CN32 was supplied by the Institute of Clean Energy and New Materials, Southwest University, China. The LB liquid medium contained 100 mL deionized water water, yeast extract 5 gL^−1^, sodium chloride 10 gL^−1^, and peptone 10 gL^−1^. LB plates were formulated by adding 16 gL^−1^ agar to the liquid medium. The M9 buffer solution contained Na_2_HPO_4_ 6 gL^−1^, KH_2_PO_4_ 3 gL^−1^, NH_4_Cl 1 gL^−1^, 1 mM MgSO_4_, 0.1 mM CaCl_2_, and NaCl 0.5 gL^−1^. The γ-Fe_2_O_3_ MNPs were obtained from Aladdin reagent, Shanghai Co., Ltd., Shanghai, China. Other chemical substances were acquired from either Aladdin Reagent Shanghai, Co., Ltd., or Shanghai McLane Biochemical Technology Co., Ltd., Shanghai, China. The experimental water was deionized (conductivity: 18.2 Ω). The culture media and their solutions were sterilized by autoclaving before use.

### 2.2. Cultivation of Heterotrophic Bacteria

The LB plates were prepared and sterilized by autoclaving at a high temperature (121 °C, 20 min). They were then quickly poured into clean culture dishes before completely cooling down. Each dish contained about 20 mL of solution and was stored in a refrigerator at 4 °C after cooling down. The preserved *S. putrefaciens* CN32 strain was retrieved from the ultra-low temperature freezer at −80 °C, activated on a solid medium, and continued to cultivate in a constant-temperature incubator at 30 °C for 14 h. It was taken out and stored in the refrigerator at 4 °C for later use. In a super-clean workbench, a single colony of *S. putrefaciens* CN32 strain grown on a solid medium was picked and put into the liquid medium after high-temperature sterilization. The conical bottle was placed on a shaker (220 rpm, 30 °C, 16.5 h) for incubation. Then, it was distributed into 6 centrifuge tubes and centrifuged (6 min, 6000 rpm) to remove the supernatant. The resulting bacteria were resuspended in 18 mM lactic acid and 80 mL of M9 buffer as the anolyte and blown with nitrogen for 30 min to ensure that the experiment was performed under strict anaerobic conditions. The obtained bacteria were named CN32. CN32@γ-Fe_2_O_3_ heterotrophic bacteria were also prepared using a similar method, wherein 7.5 mM of γ-Fe_2_O_3_ MNPs was added at the same time as the single colony. A mixture of 2 mg of γ-Fe_2_O_3_ MNPs and 20 μL of polytetrafluoroethylene (PTFE) was applied evenly on the surface of the carbon cloth (CC, 1 × 1 cm) and dried in a vacuum drying oven (110 °C, 3 h) for use as a functionalized electrode, denoted as CC@γ-Fe_2_O_3_. Then, it was used with *S. putrefaciens* CN32 in the BES system and named CN32 CC@γ-Fe_2_O_3_.

### 2.3. Application of Magnetic Fields and Data Acquisition

The schematic diagram of the electromagnetic field-coupled MFC device (Figure S1). The MFC half-cell device was placed in the coil of an integrated (SPG-03) high-frequency induction heating device. The industrial chiller was started and the magnetic field intensity was adjusted to 1–2 mT. For the i-t Curve (IT) test, intermittent magnetic stimulation was used with a time interval of 1 h, and magnetic stimulation was repeated 3 times. Continuous magnetic field application was used for electrochemical impedance spectroscopy (EIS), differential pulse voltammetry (DPV), and cyclic voltammetry (CV) testing until the test was completed. The three-electrode system of the single-chamber MFC was connected to an electrochemical workstation (CHI660 or CHI1040), with 1 × 1 cm carbon cloth (CC), saturated calomel electrode (SCE), and 2 × 2 cm CC serving as the working electrode, reference electrode, and counter electrode, respectively. The other end was connected to a computer to collect electrochemical data. CN32, CN32@γ-Fe_2_O_3_, and CC@γ-Fe_2_O_3_ were used as bioanodes for MFC systems coupled with a magnetic field, denoted as CN32 + MF, CN32@γ-Fe_2_O_3_ + MF, and CN32 CC@γ-Fe_2_O_3_ + MF, respectively.

### 2.4. Bacterial Characterization and Pretreatment

The cells were fixed in a 4% paraformaldehyde solution for 12 h and then dehydrated with a gradient of anhydrous ethanol, ranging from 30% to 100%, to ensure the complete morphology of the bacteria. The soaking time was 30 min, and after the bacterial liquid was centrifuged, the upper layer of the liquid was removed, and the sample was subjected to freeze-drying. The bacterial morphology was meticulously examined using cutting-edge techniques, including Field Emission Scanning Electron Microscopy (FESEM) and Transmission Electron Microscopy (TEM). FESEM was performed at an operating voltage of 10 kV, while TEM was conducted at an impressive operating voltage of 200 kV. The properties and content of the surface elements of the material were analyzed using Energy Dispersive Spectroscopy (EDS).

### 2.5. Electrochemical Testing of the S. putrefaciens CN32-Magnetic Field Coupling System

CV was conducted in the voltage range of −0.8 to 0.6 V relative to SCE, with a scan rate of 1–100 mV s^−1^. DPV was performed in the voltage range of −0.9 to 0.7 V with a potential increment of 0.004 V. EIS was rigorously carried out over a wide frequency range spanning from 0.1 Hz to 100 kHz. A voltage of −0.45 V was applied, accompanied by a perturbation signal of 10 mV, ensuring precise and accurate measurements. IT was measured under a constant voltage of 0.2 V to analyze the electrochemical oxidation–reduction reaction occurring at the electrode interface.

## 3. Results and Discussion

### 3.1. Assembly of CN32@γ-Fe_2_O_3_

**Figure 2:**
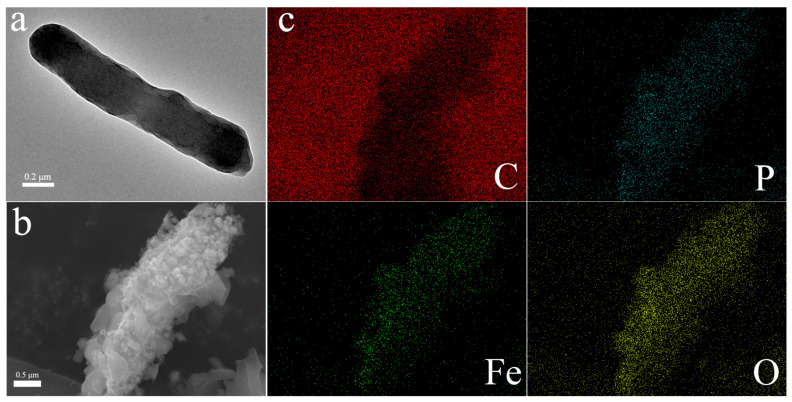
(**a**) TEM image of the unmodified *S. putrefaciens* CN32. SEM image (**b**) and EDS mapping images (**c**) of *S. putrefaciens* CN32 modified with γ-Fe_2_O_3_ MNPs.

To demonstrate the feasibility of the hybridization of the magnetic nanomaterial γ-Fe_2_O_3_ with microorganisms, a morphology analysis of CN32 and CN32@γ-Fe_2_O_3_ was performed using SEM and TEM. Figure S2 presents the X-ray diffraction pattern of the modified material γ-Fe_2_O_3_ nanoparticles. The structure reveals characteristic diffraction peaks at 2θ = 30.2, 35.5, 43.2, 53.7, 57.3, and 62.8 corresponding to the (220), (311), (400), (422), (511), and (440) crystal planes. These planes align with the standard JCPDS No. 00-039-1346 crystal structure features [44], confirming the material’s identity as γ-Fe_2_O_3_. By comparing (Figure 2a,b), it can be seen that the unmodified *S. putrefaciens* CN32 was rod-shaped and had a smooth surface. After γ-Fe_2_O_3_ MNPs were added for hybrid cultivation, the bacteria surface became rough due to the particle encapsulation, indicating the successful hybridization of γ-Fe_2_O_3_ MNPs on the surface of the bacteria. As shown in Figure 2c, the elemental analysis results clearly indicate that elements C, P, Fe, and O were uniformly distributed on the surface of the bacteria, further confirming the successful preparation of CN32@γ-Fe_2_O_3_. Corresponding data on the elemental composition of the surfaces are presented in Figure S3. The surface elemental analysis data reveal the presence of carbon (C), oxygen (O), iron (Fe), and phosphorus (P), with C and P attributed to bacterial surface constituents and Fe and O indicative of γ-Fe_2_O_3_ modifications on the surface.

### 3.2. Exploration of the Optimal Coating Amount of γ-Fe_2_O_3_ MNPs

**Figure 3:**
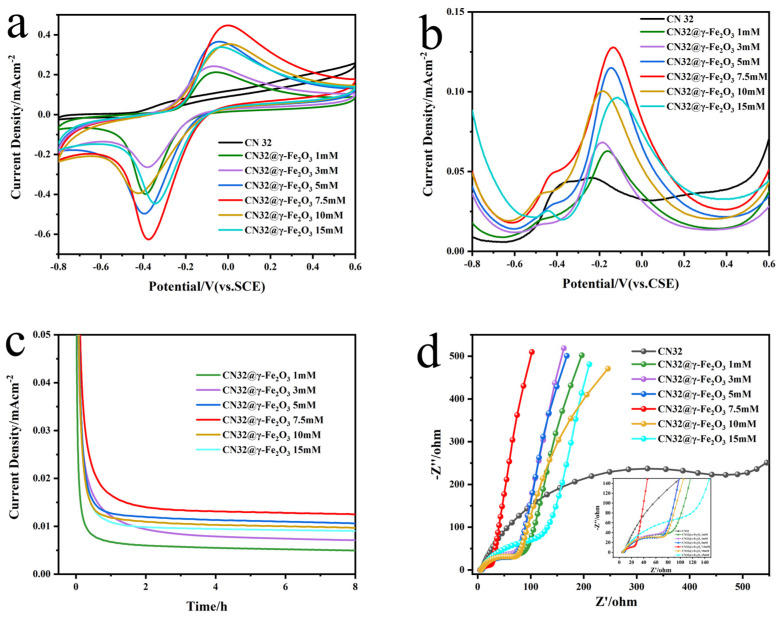
CV curve ((**a**), the scan rate is 10 mV s^-1^), DPV curve (**b**), IT curve (**c**), and EIS curve (**d**) of Natural *S. putrefaciens* CN32 and different coating concentrations of CN32@γ-Fe_2_O_3_. The coating concentrations of CN32@γ-Fe_2_O_3_ are 1 mM, 3 mM, 5 mM, 7.5 mM, 10 mM, and 15 mM, respectively.

To further investigate the effect of the surface modification of γ-Fe_2_O_3_ MNPs on bacterial bioelectrocatalytic and to identify the optimal coating amount, we assembled half-cells with different CN32@γ-Fe_2_O_3_ bioanodes, further conducted CV, DPV, EIS, and IT electrochemical tests. Figure 3a reveals the lack of a discernible redox peak in the CV curve of the naturally occurring *S. putrefaciens* CN32 strain, and only a weak redox peak at approximately −0.45 V (vs. SCE) is observed, which can be attributed to the presence of endogenous electron mediators. It is worth noting that an obvious reversible redox peak pair appears at around −0.4 V and 0 V (vs. SCE) of heterozygous bacteria at all concentrations, which correspond to the endogenous electron mediators and outer membrane C-type cytochrome protein response, respectively. Interestingly, from the CV analysis, it is evident that the oxidation peak current value exhibits a notable upward trend with an increase in the coating amount. This trend reaches its zenith at a coating amount of 7.5 mM γ-Fe_2_O_3_ MNPs, after which it gradually diminishes. At the same time, it can be observed that the cathodic and anodic peak separation is minimal for CN32@γ-Fe_2_O_3_ + 7.5 mM, indicating a faster electrochemical reaction. γ-Fe_2_O_3_ MNPs may serve as efficient electron conduits, facilitating bridging the gap for electron transfer both intra- and inter-cellularly, thus overcoming the limitations associated with long-distance electron transmission and enhancing extracellular electron transfer efficiency [45]. However, high concentrations of MNPs will decrease the electrocatalytic activity of electricity-producing bacteria, inhibiting their growth and metabolic activity.

In addition, the DPV of CN32@γ-Fe_2_O_3_ had a clear peak around −0.1 V (Figure 3b), which can be attributed to the OM c-Cyts, and the peak current density was higher than that of the undecorated bacteria, which was consistent with the trend of the CV curve. The hybrid bacteria with 7.5 mM γ-Fe_2_O_3_ MNPs had the highest electrocatalytic response. However, there was not much change in the oxidation peak attributed to self-secreted electron mediators around −0.45 V, which meant that the magnetic nanomaterials main enhanced DET mediated by OM c-Cyts, probably by improving the conductivity through the encapsulation of γ-Fe_2_O_3_ MNPs on bacterial surfaces, constructing a long-distance electron transport channel, and creating an efficient conductive network together inside and outside the biofilm. As expected, the fact that 7.5 mM was the optimal coating concentration was confirmed in the experiment. From Figure 3c, it can be seen that CN32@γ-Fe_2_O_3_ 7.5 mM had the highest stable current, which was due to the magnetic nanoparticles promoting the growth and enrichment of EAMs, expediting the growth of a biofilm on the electrode surface, and improving the output current.

Through the study of EIS, which was used to evaluate the conductivity of bioanodes [46], it was found that all the electrodes had similar impedance spectra, composed of a distinct semicircle and a straight line in Figure 3d. At the electrode–electrolyte interface, the diameter of the semicircle in the impedance spectrum represented the electron transfer resistance (*R_ct_*). A smaller *R_ct_* value indicated a greater efficiency in electron transfer, corresponding to a faster rate of electron transfer at the interface. The wild-type *S. putrefaciens* CN32 had the highest charge-transfer resistance, indicating the poor electrical conductivity of the native bacterial cells. After modification with γ-Fe_2_O_3_ MNPs, *R_ct_* was significantly reduced because of the improved conductivity of EAMs, which was beneficial for enhancing the power generation performance of MFCs. Therefore, we chose 7.5 mM γ-Fe_2_O_3_ MNPs as the optimal coating concentration to enhance the CN32 electrocatalytic activity and improve the EET efficiency. The following experiments used CN32@γ-Fe_2_O_3_ prepared by 7.5 mM γ-Fe_2_O_3_ MNPs hybridization for research.

### 3.3. Effect of EMF on Functionalized CN32@γ-Fe_2_O_3_

**Figure 4:**
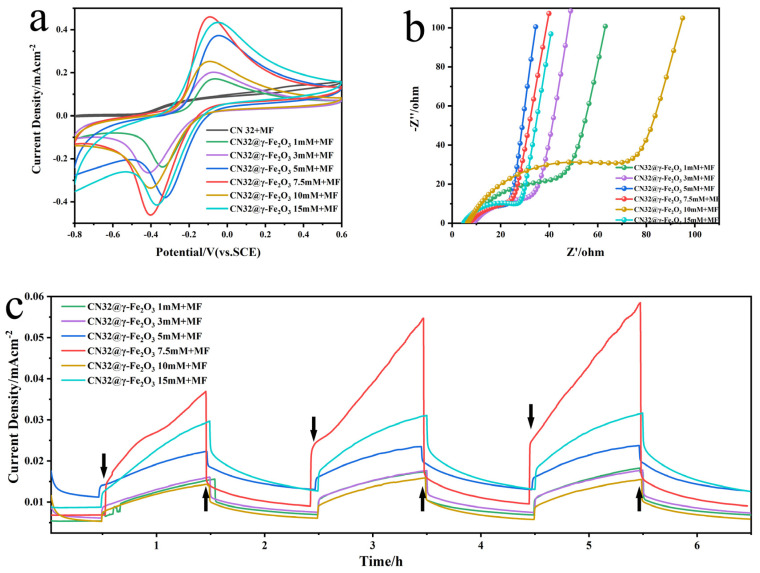
CN32@γ-Fe_2_O_3_ + MF electrochemical tests. (**a**) CV curve with a scan rate of 5 mV s^-1^. (**b**) EIS curve. (**c**) IT curve. The downward arrow indicates an opening EMF, while the upward arrow indicates a closing EMF.

Further, to explore the impact of EMF on bioelectrocatalysis, we applied the same field strength EMF to CN32@γ-Fe_2_O_3_ doped with different concentrations and carried out CV, EIS, and IT electrochemical test analyses. As seen in Figure 4a, the CN32@γ-Fe_2_O_3_ + MF redox peak currents were both substantially increased compared to the natural *S. putrefaciens* CN32 and showed reversible redox curves. CN32 CC@γ-Fe_2_O_3_ 7.5 mM + MF electrocatalytic activity was the highest. The electrocatalytic activity of the electroproducing bacteria may have been enhanced due to the stimulation of expression of OM c-Cyts and changes in oxidoreductase activity, which could be attributed to the presence of γ-Fe_2_O_3_/EMF. Figure 4b demonstrates that EMF has the capability to decrease the charge transfer resistance, thus enhancing the extracellular electron transfer capacity of electroactive bacteria. Notably, the current density exhibited a progressive increase over time, which corresponds to the process of bacteria enrichment on the electrode surface leading to the formation of a biofilm (Figure 4c). Meanwhile, CN32 CC@γ-Fe_2_O_3_ 7.5 mM + MF has the largest amount of power production, indicating that EMF can increase the specific enrichment of electroproducing bacteria at the anode. Interestingly, we also found that the effect of the applied EMF on the MFC was transient and reversible, with the current rising immediately when EMF was switched on, gradually decreasing when EMF was switched off, and gradually increasing with respect to the previous cycle during the three EMF stimulation cycles. This suggests that the EMF stimulation has a superimposed effect on MFCs, and the effect on the interior of the electroproducing bacteria is sustained, which ultimately promotes electron transfer and current generation, which is of great significance in revealing the mechanism of EET. The observed trend in the aforementioned test results aligns closely with the findings depicted in Figure 2, demonstrating an overall consistency.

### 3.4. The Mechanism of MNPs and EMF Synergistically Enhance the DET Process

**Figure 5:**
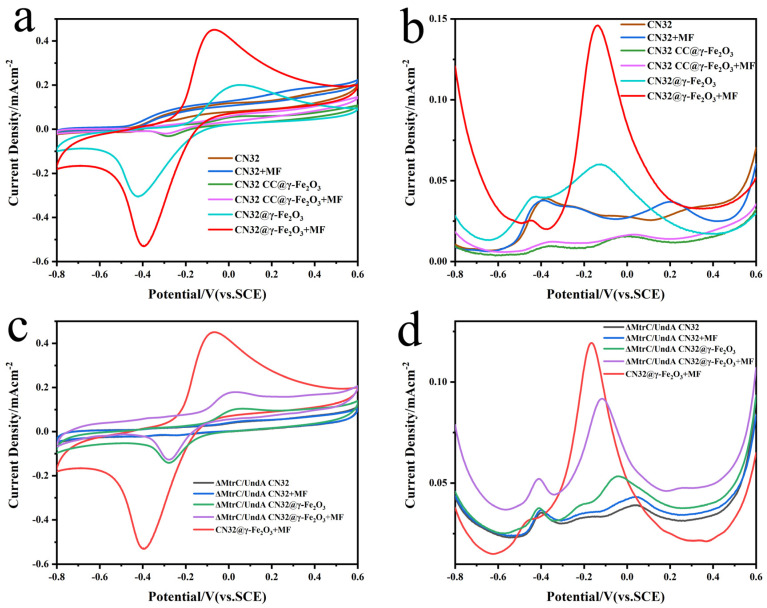
Results of electrochemical behavior tests of γ-Fe_2_O_3_ MNPs and EMF for *S. putrefaciens* CN32 and deletion bacteria ∆MtrC/UndA CN32. (**a**,**c**) CV curve with a scan rate of 10 mV s^−1^. (**b**,**d**) DPV curve.

Further investigation was conducted to explore the mechanism of hybrid bacteria coupled with EMF to synergistically promote EET. As shown in Figure 5a, CN32@γ-Fe_2_O_3_ exhibited a significant improvement in the oxidation–reduction peak current compared to CN32 or CN32 CC@γ-Fe_2_O_3_. The main reason was that the γ-Fe_2_O_3_ MNPs modified on the bacterial surface had a strong interaction with the electrogenic bacteria, thereby enhancing microbial activity, in contrast to anode electrode modification [47]. It is worth noting that the current density of the CN32 and CN32 CC@γ-Fe_2_O_3_ coupled electromagnetic field was only slightly increased. However, the oxidation peak current of CN32@γ-Fe_2_O_3_ + MF was 2.24 times higher than that of CN32@γ-Fe_2_O_3_, at 0.451 mA cm^−2^ and 0.201 mA cm^−2^, respectively. This not only indicated that the surface modification of the bacteria was beneficial to the improvement of the electrocatalysis but also suggested that the EMF played a synergistic role in promoting extracellular electron transfer mediated by γ-Fe_2_O_3_ MNPs coated on the bacterial surface. On the other hand, the oxidation–reduction peak potential of CN32@γ-Fe_2_O_3_ was −0.421 V and 0.053 V (vs. SCE), while that of CN32@γ-Fe_2_O_3_ + MF was −0.394 V and −0.07 V. The peak-to-peak distance of CN32@γ-Fe_2_O_3_ + MF was reduced by 0.15 V (0.324 V vs. 0.474 V). The reduction in peak-to-peak distance also demonstrated that MNPs and EMF synergistically accelerated EET. To further explore the interfacial redox kinetics, we tested the CV curves of different bioanodes at various scan rates, as shown in Figure S4. In the results of CN32@γ-Fe_2_O_3_ and CN32@γ-Fe_2_O_3_ + MF bioanodes, we found that the oxidation–reduction peak current and the square root of the scan rate showed a great linear relationship (5–100 mV s^−1^), indicating that diffusion control dominated the reaction process.

As shown in Figure 5b, the DPV curves of CN32@γ-Fe_2_O_3_ and CN32@γ-Fe_2_O_3_ + MF exhibit two peaks in the range of −0.8 V to 0.6 V. The peak at around −0.45 V is attributed to flavins, while the peak at around −0.1 V is related to outer membrane cytochrome protein-mediated DET. It is noteworthy that the anodic peak current density of CN32@γ-Fe_2_O_3_ reaches 0.06 mA cm^−2^, while the curve of CN32@γ-Fe_2_O_3_ + MF shows a peak current of 0.146 mA cm^−2^, which is 2.43 times higher than without the magnetic field. Correspondingly, the peak potential of CN32@γ-Fe_2_O_3_ + MF exhibits a negative shift (−0.136 V vs. −0.12 V). Moreover, it is observed that the peak of a direct electron transfer for CN32 CC@γ-Fe_2_O_3_ and CN32 CC@γ-Fe_2_O_3_ + MF is not enhanced. This may be attributed to the fact that the heterogeneous coupled electromagnetic field dual system enhances the direct electron transfer in two ways: on the one hand, γ-Fe_2_O_3_ MNPs on the bacterial surface act as electron transport channels to improve EET efficiency; on the other hand, the magnetic field promotes the specific enrichment of electrogenic bacteria, which produce additional magnetic electrons (electrons produced by magnetic-field-stimulated microorganisms) and transfer electrons through the newly formed magnetic channel, significantly enhancing the kinetics of electrochemical reactions and the efficiency of a direct electron transfer. As depicted in Figure S5, using EIS to study interfacial charge transfer behavior, CN32 modified with magnetic nanoparticles exhibited a lower *R_ct_* compared to the wild-type CN32. Further application of the EMF resulted in smaller charge transfer resistance, indicating that MNPs and EMF enhanced the conductivity of the bioanode, which is more conducive to extracellular electron transfer and the construction of a well-established biological and non-biological EET interface.

It was shown that MtrC and UndA are important OM c-Cyts in the EET process of *S. putrefaciens* CN32 [48]. In order to further investigate the mechanism of electroactive bacterial EET, the electrocatalytic properties of MNPs and EMFs for the deletion of bacterium ∆MtrC/UndA CN32 were tested. No significant oxidative reduction current was observed for ∆MtrC/UndA CN32 and ∆MtrC/UndA CN32 + MF. It may be because the deletion of the major external receptor protein of *S. putrefaciens* CN32 hinders the original EET, and a large number of electrons are unable to carry out the normal EET process. Notably, after the MNPs encapsulated the deletion bacteria, the cyclic voltammetry results showed that ∆MtrC/UndA CN32@γ-Fe_2_O_3_ and ∆MtrC/UndA CN32@γ-Fe_2_O_3_ + MF still exhibited reversible redox peaks, but the peak currents and integral areas were significantly weaker than those of CN32@γ-Fe_2_O_3_ + MF. This may be the result of electron transfer mediated by OM c-Cyts other than MtrC/UndA. The demonstrated reversible extracellular electron transfer proves that OM c-Cyts MtrC/UndA is the major mediating protein because γ-Fe_2_O_3_ MNPs can act as terminal electron acceptors to promote EET, but the absence of OM c-Cyts (UndA and MtrC) cuts off the major respiratory chain of electron transfer. Meanwhile, ∆MtrC/UndA CN32@γ-Fe_2_O_3_ + MF has greater electrocatalytic activity compared to ∆MtrC/UndA CN32@γ-Fe_2_O_3_. This result illustrates that, firstly, the binding of MNPs to outer membrane pigment proteins promotes electron transfer, and, subsequently, EMF-driven electroproducing bacteria enhance electron transfer, corroborating the synergistic promotion of EET by γ-Fe_2_O_3_ MNPs and EMF (Figure 5c).

Differential pulse voltammetry test results showed that the oxidation peak positions attributed to outer membrane pigment proteins by deletion bacteria at −0.1 V (SCE) were all positively shifted, predicting a slower electrocatalytic reaction compared to CN32@γ-Fe_2_O_3_ + MF, and the oxidation peak currents were all significantly reduced because direct electron transfer was limited by the absence of mediator proteins OM c-Cyts MtrC/UndA, which suggests a critical role for the synergistic facilitation of direct electron transfer by the γ-Fe_2_O_3_ MNPs and EMF in the synergistic promotion of DET (Figure 5d).

**Figure 6:**
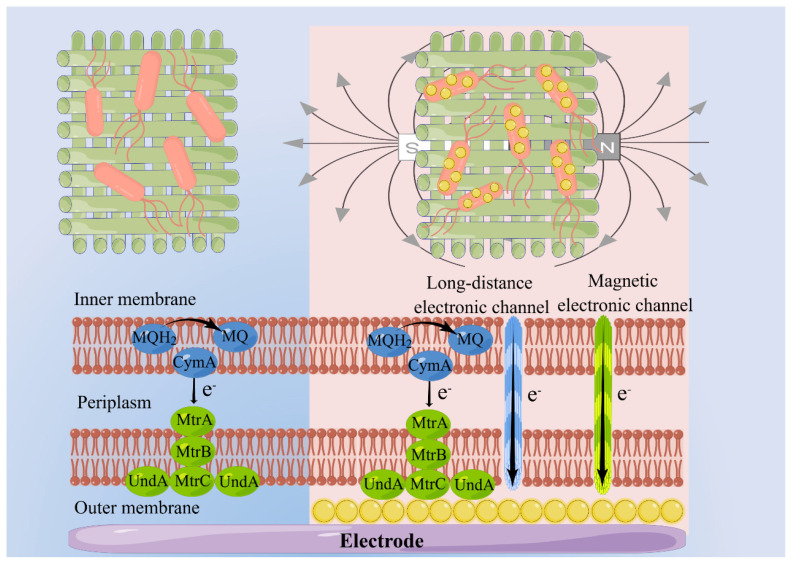
Schematic diagram illustrating the mechanism of how γ-Fe_2_O_3_ MNPs and EMFs synergistically promote EET. The downward arrow represents the direction of electron transfer.

The hypothetical mechanism of MNPs and EMF synergistically promoted EET, as shown in Figure 6. The effective interfacial electron transfer depended on close contact between the electron conduit and the receptor interface. Only the electrogenic bacteria at the outermost layer of the biofilm could perform interfacial electron transfer through direct contact. The electron transfer process at the distal end was extremely slow and may not have been fully utilized. However, when γ-Fe_2_O_3_ was coated on the surface of bacteria, acting as an electron conduit, it enhanced the conductivity of the bacteria, allowing for a direct connection between bacteria through the electron conduit. This expanded the transfer distance of electrons, resulting in the formation of an inside-out electron transfer pathway in the biological membrane. Moreover, under the influence of magnetic fluid effects caused by the applied electromagnetic field, the catalytic activity of the outer membrane cytochrome protein and metabolic reductase was improved. It opened up new magnetic channels, connecting the cytoplasm and the outer membrane for the transfer of more electrons, thus accelerating the electron transfer process. The growth metabolism of microorganisms was stimulated by the electromagnetic field, inducing cells to secrete additional magnetic electrons, thereby improving the efficiency of interfacial electron transfer in the bioelectrochemical interface.

## 4. Conclusions

In summary, a microbe hybrid system was successfully synthesized by coating γ-Fe_2_O_3_ MNPs on bacterial surface, and the bioelectrocatalytic performance of the hybrid system improved under an electromagnetic field. The γ-Fe_2_O_3_ MNPs improved the bacterial conductivity and served as an electron transport pathway for long-distance electron transfer, enhancing the efficiency of EET and the power generation performance of the MFC. In addition, the oxidation peak current of CN32@γ-Fe_2_O_3_ increased to 2.24 times under an electromagnetic field, which, due to the electromagnetic field, can greatly boost extracellular electron transfer. The enhancement mechanism is mainly due to the fact that the surface modified microorganism offers an elevated contact area for the high microbial catalytic activity of the outer cell membrane’s cytochrome, while the magnetic nanoparticles provide a networked interface between the cytoplasm and the outer membrane for boosting a fast multidimensional electron transport path under a magnetic field. This work successfully constructed a hybrid-coupled bioelectrochemical system that synergistically promotes an efficient electron transfer of MFCs, which is of great significance for increasing clean energy production using electromagnetic fields.

## Data Availability

All data needed to evaluate the conclusions in the paper are present in the paper and the Supplementary Materials.

## Supplementary Materials

The following supporting information can be downloaded at: https://www.mdpi.com/article/10.3390/ma17071501/s1, Figure S1: Schematic diagram of MFC device coupled with an electromagnetic field; Figure S2: XRD patterns of γ-Fe_2_O_3_ nanostructures; Figure S3: Data on elemental surface deposition of CN32@γ-Fe_2_O_3_ hybrid bacteria; Figure S4: The cyclic voltammogram curves of CN32@γ-Fe_2_O_3_(a) and CN32@γ-Fe_2_O_3_+MF(c) bioanodes at different scan rates. The linear regression relationship between peak current and the square root of scan rate for CN32@γ-Fe_2_O_3_(b) and CN32@γ-Fe_2_O_3_+MF(d); Figure S5: EIS curve for *S. putrefaciens* CN32, CN32@γ-Fe_2_O_3_, CN32@γ-Fe_2_O_3_+MF, CN32 CC@γ-Fe_2_O_3_, and CN32 CC@γ-Fe_2_O_3_+MF.
