# Supplementary material for: Electromagnetic Field Drives the Bioelectrocatalysis of γ-Fe2O3-Coated Shewanella putrefaciens CN32 to Boost Extracellular Electron Transfer
Source: Materials (Basel). 2024 Mar 26;17(7):1501. doi: 10.3390/ma17071501 (PMC11012369; doi:10.3390/ma17071501)
Supplement: Supplementary file 1 [file materials-17-01501-s001.zip › materials-2913832-supplementary.pdf]

materials

Supporting Information

Electromagnetic Field Drives the Bioelectrocatalysis of γ-Fe_2O_3-Coated *Shewanella putrefaciens* CN32 to Boost Extracellular Electron Transfer

Xiaohai Wang, Zhuanzhuan Shi *, Zhikai Wang and Xiaoshuai Wu *

Institute of Materials Science and Devices, School of Materials Science and Engineering, Suzhou University of Science and Technology, Suzhou 215011, China; wangxiaohai0316@163.com (X.W.); jasonwang01033@163.com (Z.W.)
* Correspondence: shizz@usts.edu.cn (Z.S.); wuxiaoshuai365@163.com (X.W.)

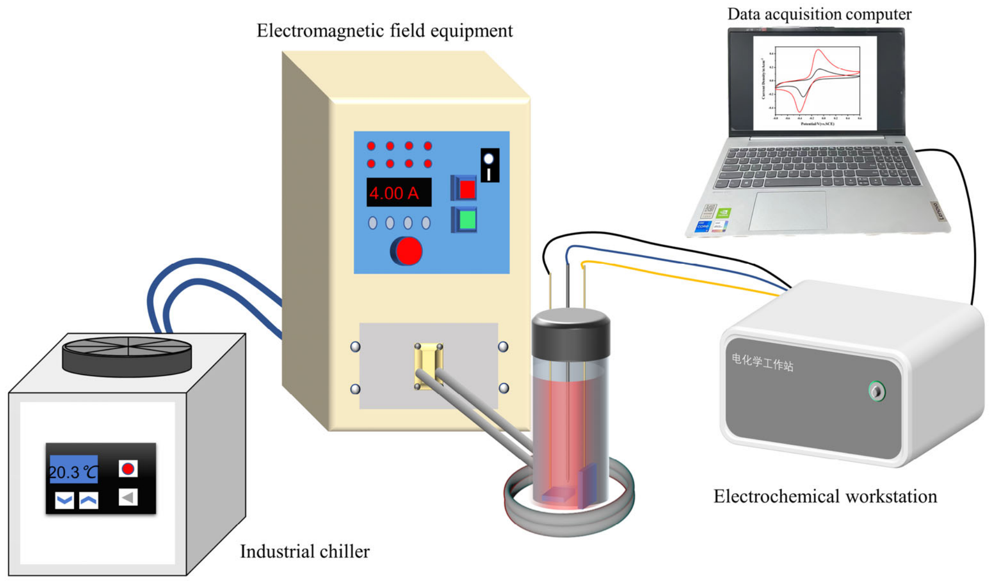

Figure S1. Schematic diagram of MFC device coupled with an electromagnetic field.

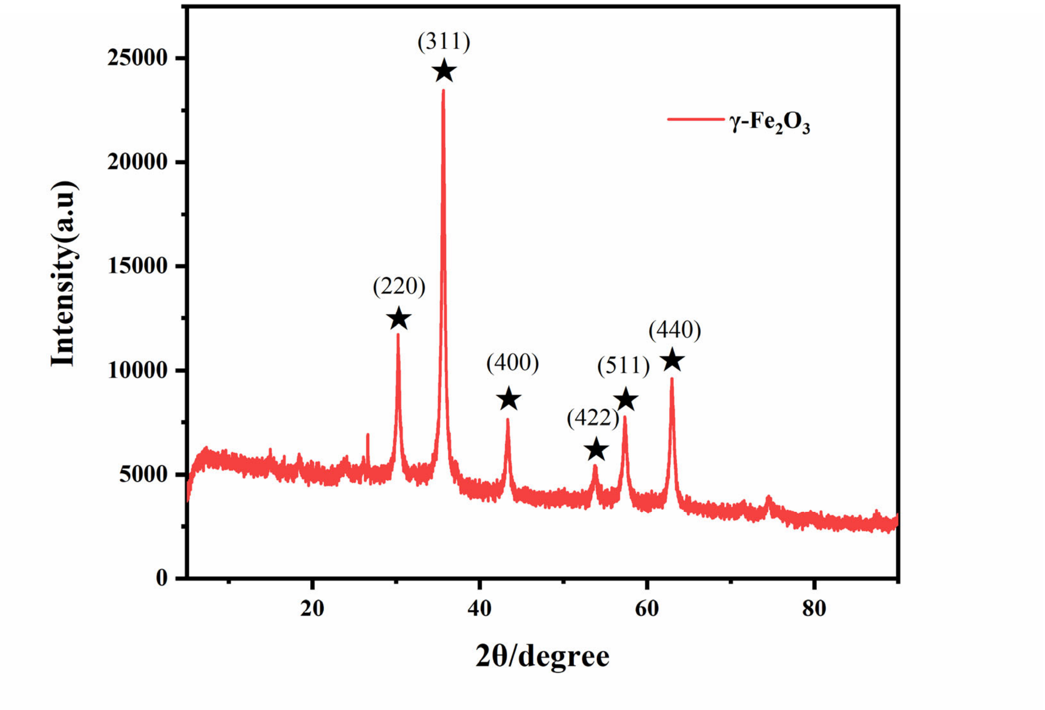

Figure S2. XRD patterns of γ-Fe_2O_3 nanostructures.

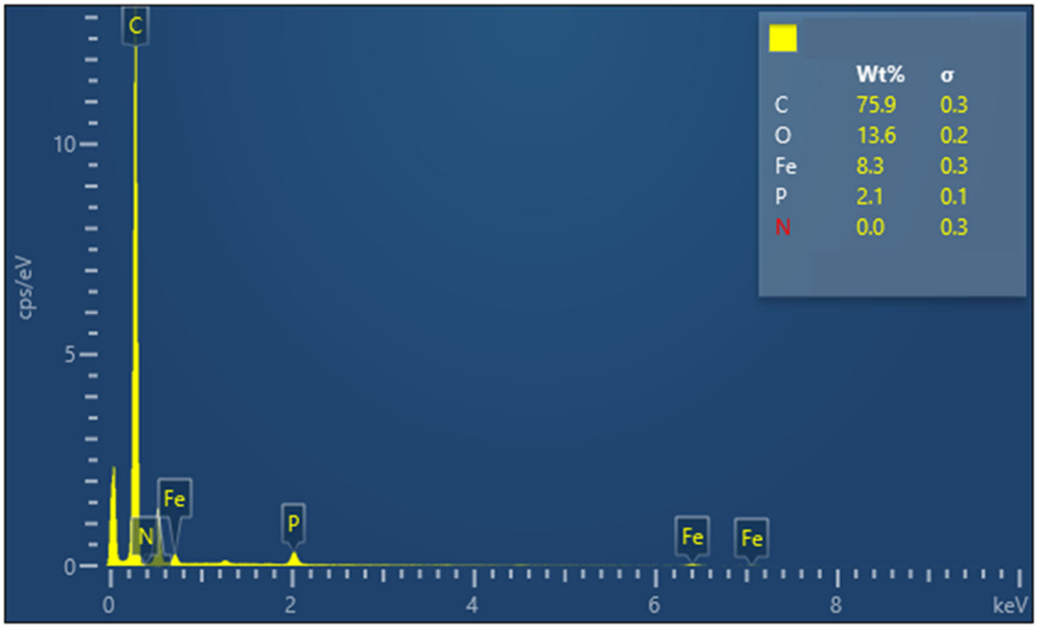

Figure S3. Data on elemental surface deposition of CN32@γ-Fe_2O_3 hybrid bacteria.

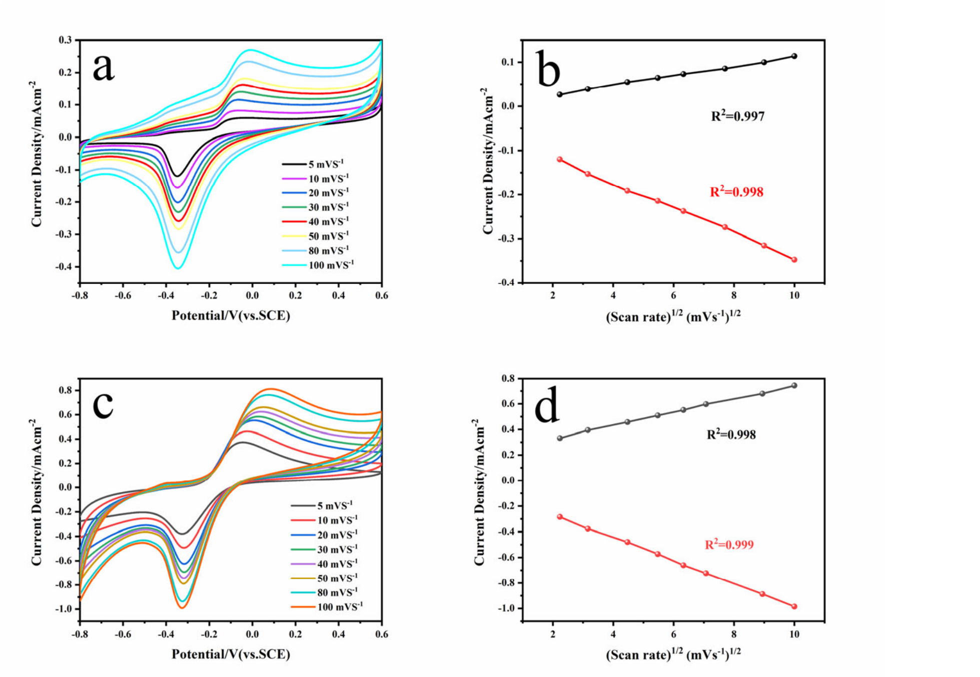

Figure S4. The cyclic voltammogram curves of CN32@γ-Fe_2O_3(a) and CN32@γ-Fe_2O_3+MF(c) bio-anodes at different scan rates. The linear regression relationship between peak current and the square root of scan rate for CN32@γ-Fe_2O_3(b) and CN32@γ-Fe_2O_3+MF(d).

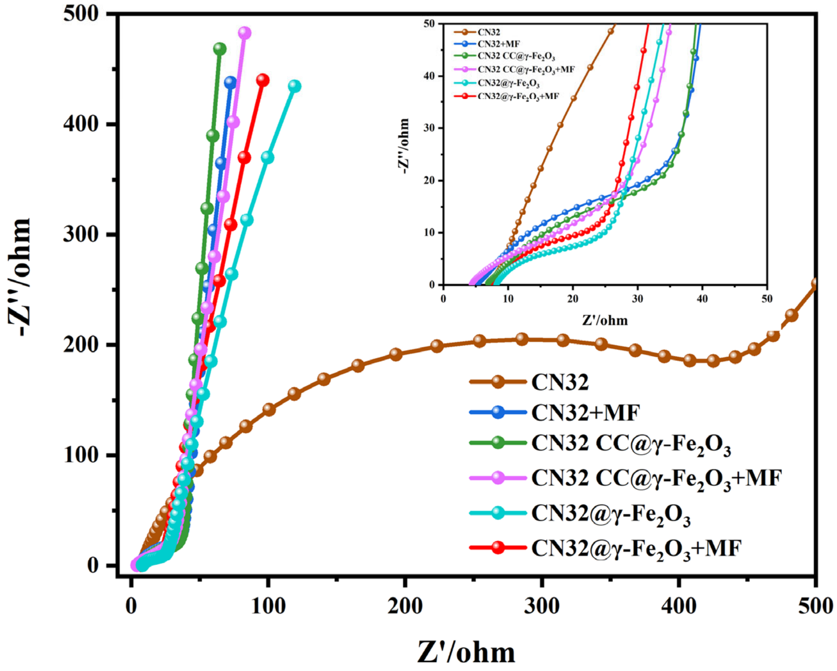

Figure S5. EIS curve for *S. putrefaciens* CN32, CN32@γ-Fe_2O_3, CN32@γ-Fe_2O_3+MF, CN32 CC@γ-Fe_2O_3, and CN32 CC@γ-Fe_2O_3+MF.
